# Baseline characteristics and prevalence of cardiovascular disease in newly visiting or referred chronic kidney disease patients to nephrology centers in Japan: a prospective cohort study

**DOI:** 10.1186/1471-2369-14-152

**Published:** 2013-07-17

**Authors:** Soichiro Iimori, Yumi Noda, Tomokazu Okado, Shotaro Naito, Takayuki Toda, Yoshiko Chida, Michio Kuwahara, Ryoichi Ando, Yasuhide Nishio, Yoshitaka Maeda, Hiroyuki Tanaka, Teiichi Tamura, Shigeaki Kimoto, Eiichiro Kanda, Seiji Inoshita, Momono Yoshikawa, Rie Okutsu, Masato Tajima, Takashi Kusaura, Katsuki Kobayashi, Tatemitsu Rai, Shinichi Uchida, Sei Sasaki

**Affiliations:** 1 Department of Nephrology, Tokyo Medical and Dental University, 1-5-45 Yushima Bunkyo-ku, Tokyo 113-8519, Japan; 2 Department of Chronic Kidney Disease, Tokyo Medical and Dental University, 1-5-45 Yushima Bunkyo-ku, Tokyo 113-8519, Japan; 3 Department of Nephrology, Tsuchiura Kyodo General Hospital, 11-7 Manabeshinmachi, Tsuchiura-shi, Ibaraki 300-0053, Japan; 4 Department of Nephrology, Nakano General Hospital, 4-59-16 Chuo, Chuo-ku, Tokyo 164-8607, Japan; 5 Department of Nephrology, Shuuwa General Hospital, 1200 Yahatashinden, Kasukabe-shi, Saitama 344-0035, Japan; 6 Department of Nephrology, Musashino Red Cross Hospital, 1-26-1 Kyonancho, Musashino-shi, Tokyo 180-8610, Japan; 7 Department of Nephrology, Tokyo Metropolitan Tama Medical Center, 2-8-29 Musashidai, Fuchu-shi, Tokyo 183-8524, Japan; 8 Department of Nephrology, JA Toride Medical Center, 2-1-1 Hongo, Toride-shi, Ibaraki 302-0022, Japan; 9 Department of Nephrology, Yokosuka Kyosai Hospital, 1-16 Yonegahamadori, Yokosuka, Kanagawa 238-8558, Japan; 10 Department of Nephrology, Ome Municipal General Hospital, 4-16-5 Higashiome, Ome-shi, Tokyo 198-0042, Japan; 11 Department of Nephrology, Tokyo Kyosai Hospital, 2-3-8 Nakameguro, Meguro-ku, Tokyo 153-8934, Japan; 12 Department of Internal Medicine, Tokyo Metropolitan Bokutoh Hospital, 4-23-15 Kohtohbashi, Sumida-ku, Tokyo 130-8575, Japan; 13 Department of Nephrology, Tokyo Metropolitan Otsuka Hospital, 2-8-1, Minamiotsuka, Toshima-ku, Tokyo 170-8476, Japan; 14 Department of Nephrology, Toshima Hospital, 33–1 Sakaecho, Itabashi-ku, Tokyo 173-0015, Japan; 15 Department of Nephrology, Tokyo Metropolitan Hiroo Hospital, 2-34-10 Ebisu, Shibuya-ku, Tokyo 150-0013, Japan; 16 Department of Nephrology, Hiratsuka Kyosai Hospital, 9-11Oiwake, Hiratsuka-shi, Kanagawa 254-8502, Japan; 17 Clinical Research Center, Chiba-East National Hospital, 673 Nitonacho, Chuo-ku, Chiba 260-8712, Japan

**Keywords:** Chronic kidney disease, Cohort study, Epidemiology, Cardiovascular disease, Nephrologist

## Abstract

**Background:**

About 39,000 patients were newly prescribed renal replacement therapy in Japan in 2011, resulting in a total of more than 300,000 patients being treated with dialysis. This high prevalence of treated end stage kidney disease (ESKD) patients is an emergent problem that requires immediate attention. We launched a prospective cohort study to evaluate population specific characteristics of the progression of chronic kidney disease (CKD). In this report, we describe the baseline characteristics and risk factors for cardiovascular disease (CVD) prevalence among this cohort.

**Methods:**

New patients from 16 nephrology centers who were older than 20 years of age and who visited or were referred for the treatment of CKD stage 2–5, but were not on dialysis therapy, were recruited in this study. At enrollment, medical history, lifestyle behaviors, functional status and current medications were recorded, and blood and urine samples were collected. Estimated glomerular filtration rate (eGFR) was calculated by a modified three-variable equation.

**Results:**

We enrolled 1138 patients, 69.6% of whom were male, with a mean age of 68 years. Compared with Western cohorts, patients in this study had a lower body mass index (BMI) and higher proteinuria. The prevalence of CVD was 26.8%, which was lower than that in Western cohorts but higher than that in the general Japanese population. Multivariate analysis demonstrated the following association with CVD prevalence: hypertension (adjusted odds ratio (aOR) 3.57; 95% confidence interval (CI) 1.82-7.02); diabetes (aOR 2.45; 95% CI 1.86-3.23); hemoglobin level less than 11 g/dl (aOR 1.61; 95% CI 1.21-2.15); receiving anti-hypertensive agents (aOR 3.54; 95% CI 2.27-5.53); and statin therapy (aOR 2.73; 95% CI 2.04-3.66). The combination of decreased eGFR and increased proteinuria was also associated with a higher prevalence of CVD.

**Conclusions:**

The participants in this cohort had a lower BMI, higher proteinuria and lower prevalence of CVD compared with Western cohorts. Lower eGFR and high proteinuria were associated with CVD prevalence. Prospective follow up of these study patients will contribute to establishment of individual population-based treatment of CKD.

## Background

Chronic kidney disease (CKD) is a major global health problem. In Japan, about 13% of the adult population, i.e. approximately 13.3 million people, are estimated to have CKD [1]. According to the National Health and Nutrition Examination Survey (NHANES 1999–2004) in the US, the reported prevalence of CKD was 13.1%, which was a substantial increase over previous reports [2]. The incidence and prevalence of end stage kidney disease (ESKD) is also rapidly increasing, with patients of ESKD having to face the problems of poor outcome and high cost of renal replacement treatment. In Japan, about 39,000 patients were introduced to renal replacement therapy in 2011, resulting in more than 300,000 patients on dialysis [3].

CKD is also a well-known risk factor for cardiovascular mortality and morbidity [4-6]. Reportedly, patients with CKD are more likely to die of cardiovascular disease (CVD) than of progression to ESKD [7]. CVD risk factors, such as hypertension, diabetes and dyslipidemia, tend to be highly prevalent and poorly controlled in patients with CKD [8]. Studies have also shown that estimated glomerular filtration rate (eGFR) and/or albuminuria are associated with cardiovascular (CV) risk [9-11]. Thus, early recognition and treatment of CKD are important to prevent CVD.

We established a large prospective cohort study of Japanese people with CKD called the CKD-ROUTE study (CKD Research of Outcomes in Treatment and Epidemiology). The specific aim of the CKD-ROUTE study is to evaluate the relationship of CKD progression, cardiovascular events and mortality to patient characteristics, complications and treatment. The findings of this study will contribute to development of appropriate therapy for CKD in the Japanese population, whose characteristics are quite different from Western populations. The study is presently ongoing, although enrollment of patients has been completed. In this report, we address whether the basic characteristics and risk factors related to CVD prevalence in Japanese patients are different from those in other ethnic groups.

## Methods

### Study design and cohort participants

The CKD-ROUTE study is a prospective, observational cohort of a representative Japanese population with CKD stages 2–5 who are not on dialysis. Over 1000 participants were enrolled at the Tokyo Medical and Dental University Hospital and its 15 affiliated, larger than mid-sized clinical centers located in the Tokyo metropolitan area of Japan, where one third of the Japanese population lives. Most patients were referred from primary care clinics. At recruitment, written informed consent was obtained from all patients and their eligibility was determined. Approval for this study was obtained from the ethics committees of all institutions participating in the study (see Acknowledgments), and the research is being conducted in accordance with the ethical principles of the Declaration of Helsinki. The protocol was registered in UMIN Clinical Trials Registry (UMIN000004461).

Participants were eligible for inclusion if they: (1) newly visited or were newly referred to the participant nephrology centers from October 2010 to December 2011; (2) were over 20 years of age; and (3) had CKD stages 2 to 5 according to Kidney Disease Improving Global Outcomes (KDIGO) classification [12]. Stage 5 CKD patients were included in this study because a recent study showed that 35% of CKD stage 5 patients did not enter renal replacement therapy over a 3-year observation period [13]. The following patients were excluded from this study: (1) patients with malignancy that was found or treated within the previous 2 years; (2) transplant recipients; (3) patients with active gastrointestinal bleeding at enrollment; and (4) patients who did not provide written informed consent. Participants have been and will be followed up at 6-month intervals. The total observation period is 3 years after enrollment or until the start of dialysis, time of death, dropout from the study or withdrawal of informed consent.

The primary study outcome is decline in renal function, defined by reduction in eGFR. Secondary outcomes are incidence of CV events, initiation of dialysis, death, hospitalization, bone fractures, and changes in the levels of blood and urine biochemical measurements, blood pressure, bodyweight and body mass index (BMI).

### Sample size

One of the major endpoints of the CKD-ROUTE study is initiation of dialysis. If the event rate of initiation of dialysis is presumed to be about 30% according to the rates shown in previous studies [13,14], in the case of 1000 subjects, the cumulative event number for the initiation of dialysis is expected to be around 300. This will have 80% power to detect a hazard ratio of 1.38 with a significant difference of 5% or less. Therefore, we considered the necessary sample size of this study as 1000 subjects.

### Measurements

At enrollment, medical history, lifestyle behaviors (self-feeding ability) and current medications were recorded. Anthropometric measurements (height and weight) were obtained and BMI was calculated. Blood pressure (BP) was measured using a standard sphygmomanometer. Blood and urine samples were collected to measure white blood cell, hemoglobin (Hb), platelet, total protein, albumin, urea nitrogen, creatinine, sodium, potassium, chloride, calcium, phosphorus, alkaline phosphatase, intact parathyroid hormone (PTH), glucose, hemoglobin A1c (HbA1c), iron, unsaturated iron binding capacity, ferritin, C-reactive protein, urinary occult blood, urinary protein, and urinary creatinine. Estimated glomerular filtration rate (eGFR) was calculated using the modified three-variable Modification of Diet in Renal Disease (MDRD) equation developed by the Japanese Society of Nephrology to adjust for Japanese physical characteristics: eGFR = 194 × serum creatinine^− 1.094^ × age^− 0.287^ (if female, × 0.739) [15]. Since the therapeutic targets of treatment of renal anemia and dyslipidemia in the Japanese CKD guidelines are Hb over 11 g/dl and LDL-cholesterol below 120 mg/dl, respectively, anemia and dyslipidemia were defined as Hb < 11 g/dl and LDL-cholesterol ≥ 120 mg/dl [16]. Corrected calcium (Ca) was adjusted for serum albumin using the equation: Ca + (4 - serum albumin), if serum albumin was < 4g/dl. As the normal range of intact PTH is 10–65 pg/ml as measured by ECLIA, high intact PTH was defined as levels over 65 pg/ml. Urinary protein to creatinine ratios (UPCR) were measured because urinary albumin is not routinely measured due to a regulation of the Japanese Medicare system, and were categorized as: optimal, UPCR < 0.15 g/gCr (gram per gram creatinine); high, UPCR 0.15-0.49 g/gCr; and very high, UPCR ≥ 0.5 g/gCr. This categorization is recommended by Japanese CKD guidelines [17]. All participants are basically treated according to the standard treatment protocols recommended by the Japanese CKD guidelines [16,17].

### Definition of hypertension, diabetes, etiology of kidney disease, and cardiovascular disease

Hypertension at entry was defined as systolic blood pressure (SBP) ≥ 140 mmHg, diastolic blood pressure (DBP) ≥ 90 mmHg, use of anti-hypertensive agents, or history of being diagnosed with hypertension. Diabetes was defined as either HbA1c ≥ 6.5% (National Glycohemoglobin Standardization Program (NGSP) method) or receiving anti-diabetic therapy. Etiology of kidney disease in each patient was determined by the physician who was treating the patient at the time of enrollment, based on patients’ past histories, clinical characteristics and findings, and histological findings in biopsied kidney specimens.

CVD was defined as a history of coronary heart disease (angina pectoris (AP), myocardial infarction (MI), or coronary revascularization), congestive heart failure (CHF), peripheral arterial disease (PAD) (necrosis, amputation or revascularization surgery), or stroke (cerebral infarction, transient ischemic attack, cerebral hemorrhage or subarachnoid hemorrhage). Prevalence of CVD at enrollment represented a composite of self-reported history of CVD or information provided by previous doctors.

### Statistical analysis

Baseline characteristics are shown as mean ± standard deviation and median with interquartile ranges for continuous variables, while categorical data are presented as numbers and percentages. Analysis of variance and chi-square test were used to compare continuous and categorical variables across CKD stages, respectively. Multivariate logistic regression analysis was used to identify the relationship between the composite CVD prevalence and predictor variables.

Statistical analyses were performed using SPSS, version 20.0. *P*-value <0.05 was considered statistically significant.

## Results

After excluding those who matched the exclusion criteria, we enrolled 1138 patients in this cohort from October 2010 to December 2011. Although we did not accurately count the number of those who were excluded in this registry, we speculate that a less than 5% of the new CKD stage 2–5 patients were excluded due to the exclusion criteria.

### Baseline demographic characteristics and prevalence of cardiovascular disease

**Figure 1:**
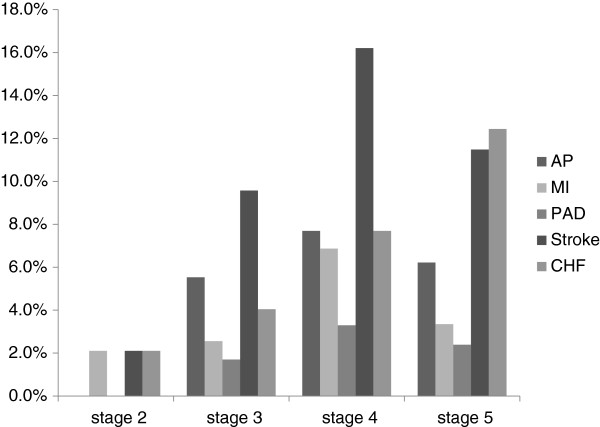
**Prevalence of individual causes of cardiovascular disease (CVD) stratified by CKD stages, the CKD-ROUTE study (Oct 2010 - Dec 2011).** Angina pectoris (AP), myocardial infarction (MI), peripheral arterial disease (PAD) and stroke were highest in patients with stage 4 CKD; congestive heart failure (CHF) incidence gradually increased with CKD progression.

The baseline demographic characteristics of the study participants are shown in Table 1. Stage 3 was most common (41.3%), followed by stage 4 and 5. Enrollment of patients with stage 2 (8.3%) is a unique feature of this cohort. Mean patient age was 68 years, and 69.6% were male. Most patients were able to feed themselves (99.1%). Mean BMI was 23.7 kg/m^2^, while 32.8% of them were overweight (BMI ≥ 25 kg/m^2^). 37.1% of the patients were diabetic. Although the percentage of patients with diabetes was significantly higher in advanced CKD stages, HbA1c was not significantly different among CKD stages. Regarding the causes of CKD, the incidence of diabetic nephropathy was higher in advanced CKD stages, while nephrosclerosis and glomerulonephritis were more frequent in moderate CKD stages. Nephrosclerosis was the overall leading cause of CKD in this cohort, through CKD stage 2–5. CVD was prevalent in 26.8% of all the patients, and patients with advanced CKD were more likely to have CVD, the prevalence being 35.7% in stage 4 and 30.6% in stage 5 patients. Regarding the individual causes of CVD, the incidence of AP, MI, PAD and stroke were highest in stage 4 patients, the incidence of CHF gradually increasing with CKD progression (Figure 1).

**Table 1:** Baseline demographics and prevalence of cardiovascular disease according to disease stage, the CKD-ROUTE study (Oct 2010 - Dec 2011)

	**All**	**Stage 2**	**Stage 3**	**Stage 4**	**Stage 5**	***P*- value**
	**n = 1138**	**n = 95**	**n = 470**	**n = 364**	**n = 209**	
Age (years old)	68 ± 14	57 ± 17	67 ± 13	71 ± 12	69 ± 14	<0.001
	70 [61–77]	61 [46–71]	70 [60–76]	73 [64–78]	70 [61–78]	
Male gender	792	57	361	255	119	<0.001
	69.6%	60.0%	76.8%	70.1%	56.9%	
Self-feeding ability	1118	94	460	360	204	0.744
	99.1%	98.9%	99.1%	99.4%	98.6%	
BMI (kg/m^2^)	23.7 ± 4.0	23.0 ± 4.0	23.7 ± 3.7	23.9 ± 4.1	23.8 ± 4.5	0.351
	23.3 [21.0 - 25.8]	22.6 [20.6 - 25.4]	23.5 [21.3 - 25.7]	23.4 [21.0 - 26.4]	23.0 [20.9 - 25.6]	
BMI ≥ 25 kg/m^2^	328	20	129	115	64	0.473
	32.8%	26.0%	32.0%	35.1%	33.2%	
Diabetes	422	15	141	163	103	<0.001
	37.1%	15.8%	30.0%	44.8%	49.3%	
HbA1c (NGSP) (%)	6.1 ± 1.0	6.0 ± 1.1	6.1 ± 1.0	6.2 ± 1.0	6.1 ± 1.0	0.083
	5.9 [5.6 - 6.5]	5.7 [5.5 - 6.1]	5.9 [5.6 - 6.4]	6.0 [5.6 - 6.6]	5.9 [5.5 - 6.5]	
Albumin (g/dl)	4.0 ± 0.8	4.0 ± 0.6	3.8 ± 0.6	3.5 ± 0.6	3.8 ± 0.6	<0.001
	4.3 [3.7 - 4.5]	4.1 [3.8 - 4.4]	3.9 [3.5 - 4.2]	3.6 [3.2 - 4.0]	4.0 [3.5 - 4.3]	
eGFR (ml/min per 1.73 m^2^)	32.7 ± 18.7	72.1 ± 8.5	43.3 ± 8.2	22.0 ± 4.4	9.9 ± 3.0	<0.001
	29.8 [17.5 - 45.0]	70.6 [64.9 - 79.7]	43.2 [35.5 - 50.2]	21.7 [17.9 - 25.6]	9.8 [7.4 - 12.4]	
SBP (mmHg)	140 ± 22	139 ± 20	137 ± 22	140 ± 24	146 ± 22	<0.001
	138 [125–152]	136 [125–150]	135 [122–150]	136 [125–154]	143 [136–160]	
DBP (mmHg)	78 ± 15	83 ± 14	79 ± 15	75 ± 15	79 ± 15	<0.001
	78 [68–87]	83 [74–92]	79 [70–87]	75 [64–83]	78 [69–90]	
Causes of CKD						
Diabetic nephropathy	287	10	74	116	87	<0.001
	25.5%	10.5%	16.0%	32.0%	42.2%	
Nephrosclerosis	451	23	214	159	55	
	40.0%	24.2%	46.2%	43.8%	26.7%	
Glomerulonephritis	216	43	83	49	41	
	19.2%	45.3%	17.9%	13.5%	19.9%	
Others	173	19	92	39	23	
	15.4%	20.0%	19.9%	10.7%	11.2%	
Prevalence of CVD	305	7	104	130	64	<0.001
	26.8%	7.4%	22.1%	35.7%	30.6%	

Continuous variables are presented as mean ± standard deviation and median with interquartile ranges. Categorical data are presented as numbers (n) of patients and percentages in each CKD stage. *BMI* body mass index, *NGSP* National Glycohemoglobin Standardization Program, *eGFR* estimated glomerular filtration rate, *SBP* systolic blood pressure, *DBP* diastolic blood pressure, *CVD* cardiovascular disease.

### CVD risk factors at enrollment

Table 2 shows the other CVD risk factors not shown in Table 1. Overall, 90.2% of the participants had hypertension at enrollment, with significantly higher BP in patients with advanced CKD stages (Table 1 and 2). The percentage of patients treated with angiotensin II receptor blockers (ARB) or angiotensin converting enzyme inhibitors (ACEI) was 63.3%, and calcium channel blockers was 42.9%. The incidence of anti-hypertensive agent usage was significantly higher in advanced CKD groups.

**Table 2:** Prevalence of non-renal risk factors for cardiovascular disease at enrollment, the CKD-ROUTE study (Oct 2010 - Dec 2011)

	**All**	**Stage 2**	**Stage 3**	**Stage 4**	**Stage 5**	***P*- value**
	**n = 1138**	**n = 95**	**n = 470**	**n = 364**	**n = 209**	
Hypertension	1027	69	406	348	204	<0.001
	90.2%	72.6%	86.4%	95.6%	97.6%	
Anti-hypertensive therapy	897	38	337	326	196	<0.001
	78.8%	40.0%	71.7%	89.6%	93.8%	
ARB or ACEI	720	29	282	271	138	<0.001
	63.3%	30.5%	60.0%	74.5%	66.0%	
Calcium channel blockers	488	13	173	180	122	<0.001
	42.9%	13.7%	36.8%	49.5%	58.4%	
β blockers	163	3	57	66	37	<0.001
	14.3%	3.2%	12.1%	18.1%	17.7%	
α blockers	76	0	22	36	18	0.001
	6.7%	0.0%	4.7%	9.9%	8.6%	
Diuretics	381	6	107	156	112	<0.001
	33.5%	6.3%	22.8%	42.9%	53.6%	
Anemia						
Hb (g/dl)	13.7 ± 1.9	13.1 ± 1.9	11.2 ± 1.9	9.8 ± 1.5	11.9 ± 2.3	<0.001
	13.9 [12.8 - 14.9]	13.2 [11.9 - 14.3]	11.1 [9.9 - 12.4]	9.8 [8.8 - 10.7]	12.0 [10.2 - 13.6]	
Hb < 11 g/dl	404	7	64	169	164	<0.001
	35.6%	7.4%	13.6%	46.6%	78.5%	
Iron deficiency	142	6	46	61	29	0.013
	14.9%	7.9%	12.2%	19.7%	15.3%	
Oral iron supplementation	58	1	12	23	22	<0.001
	5.1%	1.1%	2.6%	6.3%	10.5%	
ESA	121	1	5	48	67	<0.001
	10.6%	1.1%	1.1%	13.2%	32.1%	
Lipid abnormalities						
Total cholesterol (mg/dl)	218 ± 49	197 ± 48	185 ± 46	190 ± 51	194 ± 49	<0.001
	214 [190–248]	193 [169–216]	182 [153–217]	180 [153–220]	190 [162–220]	
HDL-C (mg/dl)	61 ± 20	55 ± 17	50 ± 16	51 ± 17	52 ± 17	<0.001
	59 [45–67]	52 [42–63]	48 [39–59]	48 [38–59]	50 [40–62]	
HDL-C < 40 mg/dl	229	11	76	89	53	<0.001
	22.5%	12.8%	18.2%	27.7%	27.7%	
LDL-C (mg/dl)	128 ± 42	111 ± 40	104 ± 39	109 ± 42	110 ± 41	<0.001
	124 [106–152]	108 [87–130]	101 [75–125]	102 [78–126]	107 [83–130]	
LDL-C ≥ 120 mg/dl	317	45	133	84	55	<0.001
	34.6%	58.4%	34.5%	29.8%	31.8%	
Statin	325	16	112	117	80	<0.001
	28.6%	16.8%	23.8%	32.1%	38.3%	
Disorders of mineral metabolism						
Corrected Ca (mg/dl)	9.6 ± 0.5	9.5 ± 0.4	9.3 ± 0.5	9.2 ± 0.9	9.4 ± 0.6	<0.001
	9.6 [9.3 -9.9]	9.5 [9.2 - 9.8]	9.3 [9.0 - 9.6]	9.2 [8.8 - 9.5]	9.4 [9.1 - 9.7]	
Corrected Ca < 8.4 mg/dl	32	1	2	8	21	<0.001
	2.9%	1.1%	0.4%	2.3%	10.2%	
P (mg/dl)	3.3 ± 0.5	3.3 ± 0.6	3.5 ± 0.6	4.6 ± 1.3	3.6 ± 0.9	<0.001
	3.4 [3.0 - 3.6]	3.3 [3.0 - 3.6]	3.5 [3.1 - 3.9]	4.4 [3.7 - 5.3]	3.5 [3.1 - 4.0]	
P ≥ 4.6 mg/dl	102	0	6	14	82	<0.001
	9.4%	0.0%	1.3%	4.0%	40.2%	
Intact PTH (pg/ml)	46 ± 22	59 ± 37	103 ± 63	246 ± 156	109 ± 108	<0.001
	39 [33–56]	51 [37–71]	89 [60–129]	218 [138–313]	71 [45–129]	
Intact PTH > 65 pg/ml	496	11	116	200	169	<0.001
	55.1%	15.9%	32.2%	68.0%	95.5%	
Urinalysis						
UPCR (g/gCr)	2.16 ± 3.23	1.40 ± 3.08	1.21 ± 2.48	2.37 ± 3.04	4.31 ± 3.99	<0.001
	0.74 [0.13 - 2.85]	0.26 [0.07 - 1.14]	0.24 [0.06 - 0.99]	1.09 [0.23 - 3.40]	3.16 [1.15 - 6.16]	
UPCR ≥ 0.5 g/gCr	591	36	159	224	172	<0.001
	56.3%	40.0%	36.7%	66.7%	90.1%	

Continuous variables are presented as mean ± standard deviation and median with interquartile ranges. Categorical data are presented as numbers (n) of patients and percentages in each CKD stage. *ARB* angiotensin receptor blockers, *ACEI* angiotensin converting enzyme inhibitors, *Hb* hemoglobin, *ESA* erythropoiesis stimulating agent, *LDL-C* low density lipoprotein cholesterol, *HDL-C* high density lipoprotein cholesterol, *TG* triglycerides, *Ca* calcium, *P* phosphorus, *PTH* parathyroid hormone, *UPCR* urinary protein to creatinine ratio, *g/gCr* gram per gram creatinine.

Anemia (Hb < 11 g/dl) was present in 35.6% of the patients, the incidence being higher in advanced CKD stages. Iron deficiency (transferrin saturation (TSAT) < 20% and ferritin < 100 ng/ml) was seen in 14.9% patients, its incidence being highest in stage 4 patients. The percentage of patients receiving oral iron supplementation and erythropoiesis stimulating agents (ESA) increased in advanced CKD groups.

A trend existed between decreasing eGFR and the increasing percentage of patients with low HDL-cholesterol. The percentage of patients with high LDL-cholesterol was higher in stage 2 and stayed relatively low through stages 3 to 5. Statin therapy was prescribed in 28.6% of patients, the percentage significantly increasing in advanced CKD groups.

Low corrected serum calcium levels (< 8.4 mg/dl) and high phosphorus levels (≥ 4.6 mg/dl) were seen in patients with stage 5 CKD. The percentage of patients with high intact PTH levels (> 65 pg/ml) was 15.9% in stage 2, 32.2% in stage 3, 68% in stage 4 and 95.5% in stage 5 CKD, respectively. On urinalysis, UPCR was significantly higher in advanced CKD stages, with 90% of stage 5 CKD patients having a UPCR over 0.5 g/gCr.

### Risk factors for the prevalence of CVD

The results of multiple logistic regression analysis of the risk factors that predict the prevalence of CVD at enrollment are shown in Table 3. Odds ratio was adjusted for age and gender. Hypertension, diabetes, advanced CKD stage and UPCR greater than 0.5 g/gCr were associated with high CVD prevalence. Receiving anti-hypertensive agents was associated with CVD prevalence. However, SBP over 130 mmHg at the first visit was not associated with CVD. Anemia (Hb < 11 g/dl), iron deficiency, use of oral iron supplementation and ESA were also associated with the prevalence of CVD. Low HDL-cholesterol and receiving lipid lowering therapy (use of statins) were associated with CVD. However, the odds ratio of LDL-cholesterol ≥ 120 mg/dl was 0.49, indicating that low LDL-cholesterol was associated with CVD risk. Hyperparathyroidism (intact PTH > 65 pg/ml) was found to be associated with CVD. However, low corrected serum calcium (< 8.4 mg/dl) and high serum phosphorus (≥ 4.6 mg/dl) were not associated with CVD risk. In addition, since we sometimes encounter patients with CHF that is unrelated to atherosclerotic diseases, we analyzed the factors associated with the prevalence of CVD that excludes CHF from the definition. All the risk factors were still significant, except for three factors, namely, anemia, use of ESA and use of oral iron supplementation (Additional file 1: Table S1).

**Table 3:** Risk factors for the presence of cardiovascular disease, the CKD-ROUTE study (Oct 2010 - Dec 2011)

	**Unadjusted OR [95% CI]**	***P*- value**	**Adjusted OR [95% CI]**	***P*- value**
Hypertension	4.07 [2.10 - 7.90 ]	<0.001	3.57 [1.82 - 7.02]	<0.001
Diabetes	2.32 [1.77 - 3.03]	<0.001	2.45 [1.86 - 3.23]	<0.001
BMI				
Optimal BMI (18.5 to 24.9 kg/m^2^)	1 (reference)		1 (reference)	
≥ 25 kg/m^2^	1.10 [0.82 - 1.49]	0.513	1.27 [0.93 - 1.74]	0.130
< 18.5 kg/m^2^	0.77 [0.43 -1.39]	0.391	0.74 [0.40 - 1.36]	0.329
CKD stage				
Stage 2	1 (reference)		1 (reference)	
Stage 3	3.57 [1.61 - 7.95]	0.002	2.56 [1.14 - 5.78]	0.023
Stage 4	6.98 [3.14 - 15.53]	<0.001	4.79 [2.12 - 10.81]	<0.001
Stage 5	5.55 [2.43 - 12.65]	<0.001	4.37 [1.89 - 10.11]	0.001
UPCR				
< 0.15 g/gCr	1 (reference)		1 (reference)	
0.15 to 0.49 g/gCr	1.21 [0.78 - 1.89]	0.402	1.23 [0.78 - 1.94]	0.378
≥ 0.5 g/gCr	1.55 [1.11 - 2.16]	0.009	1.77 [1.26 - 2.50]	0.001
				
SBP ≥ 130 mmHg	0.90 [0.68 - 1.18]	0.438	0.93 [0.70 - 1.23]	0.600
Anti-hypertensive therapy	3.92 [2.53 - 6.07]	<0.001	3.54 [2.27 - 5.53]	<0.001
Use of ARB or ACEI	1.72 [1.29 - 2.29]	<0.001	1.64 [1.22 - 2.20]	0.001
Use of calcium channel blocker	1.55 [1.19 - 2.02]	0.001	1.55 [1.18 - 2.03]	0.002
Use of diuretics	1.61 [1.08 - 2.38]	0.019	2.44 [1.84 - 3.24]	<0.001
Hb < 11 g/dl	1.63 [1.24 - 2.13]	<0.001	1.61 [1.21 - 2.15]	0.001
Iron deficiency	2.03 [1.40 - 2.94]	<0.001	2.41 [1.63 - 3.58]	<0.001
Use of ESA	2.10 [1.43 - 3.10]	<0.001	1.93 [1.29 - 2.89]	0.001
Use of oral iron supplementation	2.01 [1.17 - 3.44]	0.011	2.15 [1.22 - 3.80]	0.008
LDL-C ≥ 120 mg/dl	0.44 [0.31 - 0.61]	<0.001	0.49 [0.34 - 0.68]	<0.001
HDL-C < 40 mg/dl	2.45 [1.80 - 3.35]	<0.001	2.29 [1.66 - 3.16]	<0.001
TG ≥ 150 mg/dl	0.81 [0.61 - 1.08]	0.149	0.92 [0.69 - 1.23]	0.575
Use of statin	2.48 [1.88 - 3.27]	<0.001	2.73 [2.04 - 3.66]	<0.001
Corrected Ca < 8.4 mg/dl	0.49 [0.19 - 1.30]	0.152	0.46 [0.17 - 1.22]	0.118
P ≥ 4.6 mg/dl	1.12 [0.72 - 1.76]	0.611	1.34 [0.84 - 2.14]	0.214
Intact PTH > 65 pg/ml	1.80 [1.33 - 2.43]	<0.001	1.86 [1.36 - 2.54]	<0.001

Odds ratio (OR) was adjusted by age and gender. *CI* confidence interval, *BMI* body mass index, *UPCR* urinary protein to creatinine ratio, *g/gCr* gram per gram creatinine, *SBP* systolic blood pressure, *ARB* angiotensin receptor blockers, *ACEI* angiotensin converting enzyme inhibitors, *Hb* hemoglobin, *ESA* erythropoiesis stimulating agent, *LDL-C* low density lipoprotein cholesterol, *HDL-C* high density lipoprotein cholesterol, *TG* triglycerides, *Ca* calcium, *P* phosphorus, *PTH* parathyroid hormone.

**Figure 2:**
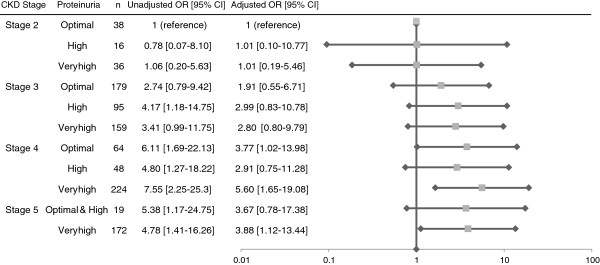
**Prevalence of cardiovascular disease (CVD) in 11 categories classified by CKD stages and proteinuria, the CKD-ROUTE study (Oct 2010 - Dec 2011).** Odds ratio (OR) was adjusted for age and gender. The reference category is the group with stage 2 and optimal proteinuria. Horizontal lines indicate 95% confidence intervals (95% CI) and the scales are logarithmic. eGFR lower than 30 ml/min per 1.73 m^2^ and very high proteinuria were significantly associated with the prevalence of CVD.

Figure 2 shows the prevalence of CVD in 11 categories classified based on CKD stages and proteinuria. Patients with stage 4 and 5 CKD with very high proteinuria were significantly associated with the prevalence of CVD.

## Discussion

The CKD-ROUTE study was established to identify the risk factors of CKD and effects of treatment on its progression. A unique characteristics of this study is that all enrolled patients are those who newly visited or were referred to our nephrology centers. This gives us information on the basic characteristics of the patients before they receive specific treatment from nephrologists, allowing clear evaluation of the efficacy of the treatment by nephrologists in a prospective manner. Although the dietary habits in Japan have been westernized, there are many differences between Japanese compared to Western populations, such as lower calorie intake, smaller body size and lower BMI [18,19]. It is highly possible that these differences affect the progression and treatment of CKD. The ROUTE study has the potential to help establish ethnic group-based CKD guidelines.

Keeping such differences in mind, at the completion of the enrollment, we addressed whether the basic characteristics of ROUTE patients are different from those of Western cohorts, such as those in the CRIC study in the US [20,21], CRISIS study in UK [22], and MERENA study in Spain [23]. As shown in Table 4, ROUTE participants were older, with a lower BMI, lower prevalence of diabetes, higher SBP and DBP, and lower prevalence of CVD. Thus, the patients in this cohort differ from Western populations in many aspects, with some of these differences probably reflecting basic physical and habitual differences among the ethnic groups.

**Table 4:** Comparison of baseline characteristics of CKD cohort studies

	**ROUTE**	**CRIC **[20]**,**[21]	**CRISIS **[22]	**MERENA **[23]
	**Japan**	**US**	**UK**	**Spain**
	**n = 1138**	**n = 3612**	**n = 1325**	**n = 1129**
eGFR (ml/min per 1.73 m^2^)	0 - 90	20 - 70	10 - 60	15 - 60
Age (years)	68	58.2	65.1	68
Male gender (%)	69.6	54	63.7	64
BMI (kg/m^2^)	23	32.1		28.4
eGFR (ml/min per 1.73 m^2^)	32.7	43.4	30.9	28
Diabetes (%)	37.1	47	32.4	40.8
Hypertension (%)	90.2	86		92.7
SBP (mmHg)	140	127.7	138.3	141
DBP (mmHg)	78	71.4	75.2	76
ARB or ACEI (%)	63	68	59.8	
Hb (g/dl)	11.9	12.7	12.41	12.8
LDL-C (mg/dl)	110	102.5		116
Ca (mg/dl)	9.1	9.2	9.14	
P (mg/dl)	3.6	3.7	3.72	3.7
Intact PTH (pg/ml)	109	53	93.2	145
Prevalence of CVD (%)	26.8	33.4	47.2	39.1
Proteinuria (mean)	2.16 g/gCr		1.08 g/day	1.2 g/day
Proteinuria (median)	0.74 g/gCr	0.17 g/day		

*BMI* body mass index, *eGFR* estimated glomerular filtration rate, *SBP* systolic blood pressure, *DBP* diastolic blood pressure, *ARB* angiotensin receptor blockers, *ACEI* angiotensin converting enzyme inhibitors, *Hb* hemoglobin, *LDL-C* low density lipoprotein cholesterol, *Ca* calcium, *P* phosphorus, *PTH* parathyroid hormone, *CVD* cardiovascular disease, *g/gCr* gram per gram creatinine.

Although the prevalence of CVD in ROUTE participants is lower than that in Western CKD cohorts (Table 4), it is still higher than that in the general Japanese population [24]. As summarized in Tables 1 and 2, many established risk factors of CVD showed a high prevalence in this cohort, with an increase in the prevalence of CVD with worsening CKD stage. We analyzed which factors could predict the prevalence of CVD at enrollment. This analysis is important because the determined factors in turn may predict the future occurrence of new CV events. As summarized in Table 3, many factors predicted the CVD prevalence at enrollment, some of which are discussed below.

As expected, hypertension and diabetes were associated with CVD prevalence. Although receiving anti-hypertensive therapy was an associated factor for CVD, SBP ≥ 130 mmHg at enrollment was not a significant predictor. This could be explained by the fact that patients with CVD were already prescribed anti-hypertensive drugs by primary care physicians, resulting in normalized BP. Interestingly, the adjusted odds ratio for the use of diuretics was higher than for other anti-hypertensive agents. A most likely explanation for this would be that participants with CVD have been prescribed diuretics for treatment of CHF or edema. Although not shown, the fact that 78.7% of the patients who experienced CHF used diuretics supports this explanation.

Increasing UPCR was also associated with the prevalence of CVD. Furthermore, as shown in Figure 2, the combination of advanced CKD stage and high proteinuria was significantly associated with CVD prevalence. It is of note that the amount of urinary protein excretion was high in ROUTE patients compared with the participants of Western CKD cohorts (Table 4). The reason for this is not immediately clear, but relatively poor control of BP may partly account for this. Also, the fact that the participants were new patients not previously treated by nephrologists, and were not given specific treatments could have contributed to this difference.

Dyslipidemia is a conventional CV risk factor that is also commonly observed in CKD patients, with an increasing incidence with progression of CKD stage [25]. Statins are the most important pharmacologic approach altering cholesterol levels, with more than one fourth of the patients in this study being treated by statins. Our data showed that although statin therapy was an associated factor for CVD, high LDL-cholesterol levels were not, suggesting that patients who had CVD were already prescribed lipid lowering drugs by primary care physicians, same as anti-hypertensive drugs.

Anemia was also an associated factor of CVD prevalence in this study. Patients with eGFR below 60 ml/min per 1.73 m^2^ are much more likely to have anemia, and the prevalence and severity of anemia increases with declining kidney function [26]. In the present study, more than almost half of the CKD stage 4 and 5 patients had anemia (Hb < 11g/dl), with an incidence of iron deficiency of 19.7% and 15.3% in these stages, respectively. However, the percentage of patients receiving oral iron or ESA therapy was only 5% and 10%, respectively, reflecting insufficient treatment by primary care physicians. Follow up of the cohort by nephrologists for adequate treatment will provide for the effect of treatment of anemia in CKD patients.

## Conclusions

The ROUTE participants were characterized by low BMI, high proteinuria and low prevalence of CVD. Hypertension, diabetes, anemia, decreasing eGFR and increasing proteinuria are associated with the prevalence of CVD. Receiving anti-hypertensive agents, statin therapy and treatment for anemia are also associated with the prevalence of CVD. Prospective follow up of ROUTE participants treated by nephrologists will provide high quality evidence of the risk factors of progression of CKD and their appropriate treatment.

## Abbreviations

ESKD: End stage kidney disease; CKD: Chronic kidney disease; CVD: Cardiovascular disease; eGFR: Estimated glomerular filtration rate; BMI: Body mass index; aOR: Adjusted odds ratio; NHANES: National health and nutrition examination surveys; CV: Cardiovascular; ROUTE: Research and outcome in treatment and epidemiology; NKF: National kidney foundation; K/DOQI: Kidney disease outcomes quality initiative; BP: Blood pressure; MDRD: Modification of diet in renal disease; Ca: Calcium; UPCR: Urinary protein to creatinine ratio; SBP: Systolic blood pressure; DBP: Diastolic blood pressure; HbA1c: Hemoglobin A1c; NGSP: National glycohemoglobin standardization program; AP: Angina pectoris; MI: Myocardial infarction; CHF: Congestive heart failure; PAD: Peripheral arterial disease; Hb: Hemoglobin; TSAT: Transferrin saturation; ESA: Erythropoiesis stimulation agent; PTH: Parathyroid hormone.

## Competing interests

The authors declare that they have no competing interests.

## Authors’ contributions

YN and SN contributed to the design of this study. SI managed the database, performed statistical analysis and drafted the manuscript. SS contributed to organizing the study and writing the manuscript. EK advised on statistical analysis. SI, YN, TO, SN, TT, YC, MK, RA, YN, YM, HT, TT, SK, EK, SI, MY, RO, MT, TK, KK, TR, SU and SS treated the patients and provided the patients’ data. All authors have read and approved the final manuscript.

## Pre-publication history

The pre-publication history for this paper can be accessed here:

http://www.biomedcentral.com/1471-2369/14/152/prepub

## Supplementary Material

Additional file 1: Table S1 Risk factors for the presence of cardiovascular disease excluding congestive heart failure, CKD-ROUTE study (Oct 2010 - Dec 2011). Click here for file
