# Supplementary material for: Baseline characteristics and prevalence of cardiovascular disease in newly visiting or referred chronic kidney disease patients to nephrology centers in Japan: a prospective cohort study
Source: BMC Nephrol. 2013 Jul 17;14:152. doi: 10.1186/1471-2369-14-152 (PMC3723419; doi:10.1186/1471-2369-14-152)
Supplement: Additional file 1: Table S1 — Risk factors for the presence of cardiovascular disease excluding congestive heart failure, CKD-ROUTE study (Oct 2010 - Dec 2011). [file 1471-2369-14-152-S1.docx]

**Suppl Table 1. Risk factors for the presence of cardiovascular disease excluding congestive heart failure, CKD-ROUTE study (Oct 2010 - Dec 2011)**

|  | Unadjusted OR [95%CI] | P-value |  | Adjusted OR [95%CI] | P-value |
| --- | --- | --- | --- | --- | --- |
| Hypertension | 4.98 [2.29 - 10.85] | <0.001 |  | 4.42 [2.01 - 9.73] | <0.001 |
| Diabetes | 2.35 [1.77 - 3.11] | <0.001 |  | 2.50 [1.87 - 3.35] | <0.001 |
|  |  |  |  |  |  |
| BMI |  |  |  |  |  |
| optimal BMI (18.5 to 24.9 kg/m^2^) | 1 (reference) |  |  | 1 (reference) |  |
| ≥ 25 kg/m^2^ | 1.09 [0.80 - 1.48] | 0.593 |  | 1.26 [0.91 - 1.73] | 0.164 |
| < 18.5 kg/m^2^ | 0.44 [0.21 - 0.90] | 0.025 |  | 0.40 [0.19 - 0.85] | 0.017 |
|  |  |  |  |  |  |
| CKD stage |  |  |  |  |  |
| stage 2 | 1 (reference) |  |  | 1 (reference) |  |
| stage 3 | 4.50 [1.78 - 11.39] | 0.001 |  | 3.19 [1.24 - 8.17] | 0.016 |
| stage 4 | 8.74 [3.46 - 22.09] | <0.001 |  | 5.95 [2.32 - 15.25] | <0.001 |
| stage 5 | 5.22 [2.00 - 13.60] | 0.001 |  | 4.09 [1.55 - 10.8] | 0.005 |
|  |  |  |  |  |  |
| UPCR |  |  |  |  |  |
| < 0.15 g/gCr | 1 (reference) |  |  | 1 (reference) |  |
| 0.15 to 0.49 g/gCr | 1.20 [0.76 - 1.89] | 0.441 |  | 1.22 [0.76 - 1.95] | 0.406 |
| ≥ 0.5 g/gCr | 1.34 [0.95 - 1.89] | 0.092 |  | 1.54 [1.08 - 2.20] | 0.017 |
|  |  |  |  |  |  |
| SBP ≥ 130 mmHg | 0.91 [0.68 - 1.22] | 0.522 |  | 0.94 [0.70 - 1.28] | 0.705 |
| Anti-hypertensive therapy | 4.15 [2.57 - 6.71] | <0.001 |  | 3.76 [2.31 - 6.13] | <0.001 |
| Use of ARB or ACEI | 1.85 [1.36 - 2.50] | <0.001 |  | 1.76 [1.29 - 2.41] | <0.001 |
| Use of calcium channel blocker | 1.71 [1.30 - 2.26] | <0.001 |  | 1.73 [1.30 - 2.31] | <0.001 |
| Use of diuretics | 1.90 [1.43 - 2.52] | <0.001 |  | 1.86 [1.39 - 2.50] | <0.001 |
|  |  |  |  |  |  |
| Hb < 11 g/dl | 1.34 [1.01 - 1.77] | 0.044 |  | 1.31 [0.97 - 1.78] | 0.077 |
| Iron deficiency | 1.95 [1.34 - 2.86] | 0.001 |  | 2.38 [1.59 - 3.58] | <0.001 |
| Use of ESA | 1.59 [1.06 - 2.40] | 0.026 |  | 1.44 [0.94 - 2.20] | 0.096 |
| Use of oral iron supplementation | 1.52 [0.85 - 2.69] | 0.154 |  | 1.61 [0.88 - 2.94] | 0.122 |
|  |  |  |  |  |  |
| LDL-C ≥ 120 mg/dl | 0.42 [0.29 - 0.60] | <0.001 |  | 0.47 [0.33 - 0.68] | <0.001 |
| HDL-C < 40 mg/dl | 2.39 [1.74 - 3.29] | <0.001 |  | 2.20 [1.58 - 3.07] | <0.001 |
| TG ≥ 150 mg/dl | 0.84 [0.62 - 1.13] | 0.240 |  | 0.96 [0.71 - 1.31] | 0.800 |
| Use of statin | 2.82 [2.11 - 3.76] | <0.001 |  | 3.21 [2.37 - 4.35] | <0.001 |
|  |  |  |  |  |  |
| Corrected Ca < 8.4 mg/dl | 0.45 [0.16 - 1.31] | 0.144 |  | 0.41 [0.14 - 1.21] | 0.107 |
| P ≥ 4.6 mg/dl | 0.61 [0.36 - 1.05] | 0.075 |  | 0.72 [0.41 - 1.25] | 0.247 |
| Intact PTH > 65 pg/ml | 1.56 [1.14 - 2.13] | 0.005 |  | 1.62 [1.17 - 2.24] | 0.003 |

Odds ratio (OR) was adjusted by age and gender. CI, confidence interval; BMI, body mass index; UPCR, urinary protein to creatinine ratio; g/gCr, gram per gram creatinine; SBP, systolic blood pressure; ARB, angiotensin receptor blockers; ACEI, angiotensin converting enzyme inhibitors; Hb, hemoglobin; ESA, erythropoiesis stimulating agent; LDL-C, low density lipoprotein cholesterol; HDL-C, high density lipoprotein cholesterol; TG, triglycerides; Ca, calcium; P, phosphorus; PTH, parathyroid hormone.
